# Microbiological Culture Simplified Using Anti-O12 Monoclonal Antibody in TUBEX Test to Detect *Salmonella* Bacteria from Blood Culture Broths of Enteric Fever Patients

**DOI:** 10.1371/journal.pone.0049586

**Published:** 2012-11-16

**Authors:** Jusak Nugraha, Ferdy R. Marpaung, Frankie C. H. Tam, Pak Leong Lim

**Affiliations:** 1 Department of Clinical Pathology, Faculty of Medicine, Airlangga University/Dr. Soetomo Hospital, Surabaya, Indonesia; 2 Tropical Disease Center, Airlangga University, Surabaya, Indonesia; 3 IgGENE, Fotan, Hong Kong; Indian Institute of Science, India

## Abstract

Definitive diagnosis of infectious diseases, including food poisoning, requires culture and identification of the infectious agent. We described how antibodies could be used to shorten this cumbersome process. Specifically, we employed an anti-*Salmonella* lipopolysaccharide O12 monoclonal antibody in an epitope-inhibition 10-min test (TUBEX TP) to detect O12^+^ *Salmonella* organisms directly from routine blood culture broths. The aim is to obviate the need to subculture the broth and subsequently identify the colonies. Thus, blood from 78 young outpatients suspected of having enteric fever was incubated in an enrichment broth, and after 2 or 4 days, broth samplings were examined by TUBEX TP as well as by conventional agar culture and identification. TUBEX TP was performed before the culture results. Eighteen isolates of *S*. Typhi (15 after 2 days) and 10 isolates of *S*. Paratyphi A (4 after 2 days) were obtained by conventional culture. Both these *Salmonella* serotypes, the main causes of enteric fever, share the O12 antigen. In all instances where either of these organisms was present (cultured), TUBEX TP was positive (score 4 [light blue] – to – score 10 [dark blue]; negative is 0 [pink-colored]) i.e. 100% sensitive. Identification of the specific *Salmonella* serotype in TUBEX-positive cases was achieved subsequently by conventional slide agglutination using appropriate polyclonal antisera against the various serotypes. Twelve *Escherichia coli*, 1 *Alcaligenes* spp. and 1 *Enterobacter* spp. were isolated. All of these cases, including all the 36 culture-negative broths, were TUBEX-negative i.e. TUBEX TP was 100% specific. In a separate study using known laboratory strains, TUBEX TF, which detects *S*. Typhi but not *S*. Paratyphi A via the O9 antigen, was found to efficiently complement TUBEX TP as a differential test. Thus, TUBEX TP and TUBEX TF are useful adjuncts to conventional culture because they can save considerable time (>2 days), costs and manpower.

## Introduction

It is important that infectious diseases are correctly identified as quickly as possible for the sake of both patient care and public health. This, however, is sometimes difficult when only clinical evidence is available, because many diseases of different origins can resemble one another. An example is the acute fevers seen commonly in the tropics, which include not only enteric fever, but also dengue fever, malaria and rickettsial fever among the host of diseases. Another example is food poisoning that affects both resource-poor and developed economies [1], which is caused, commonly, by several types of bacteria and viruses. A recent outbreak due to enterohemorrhagic *Escherichia coli* which plagued Europe for weeks [2] underscores just how vulnerable the global community (or economy) is to such infections.

Definitive diagnosis relies on good laboratory investigations. For many diseases, either or both of the following investigative approaches are usually adopted: (a) Isolation and identification of the infectious agent in culture media, and (b) Detection of antigens of the infectious agent, or antibodies induced by these antigens, in the serum or other body fluids of the infected patient. Which one of these approaches is more appropriate or efficacious depends on the disease. For example, at the extremes, culturing is always used to investigate cases of food poisoning, while serology (based on immunological detection) is almost exclusively used to diagnose syphilis. Traditionally, however, culturing is often regarded as the gold standard of diagnosis, a view that has recently been questioned for some diseases [3], [4]. The main problem with culturing is that this is often long and cumbersome, and it requires a specialized laboratory and staff. Serology, on the other hand, allows a faster turnaround time and is generally less demanding on personnel and laboratory. In fact, great strides have been made over the years in immunodiagnosis with the introduction of point-of-care tests that do not require laboratory or instrumentation, and which can be performed by non-specialist staff. Importantly, the results of many of these tests can be known within 10 mins. In contrast, little progress has been made to make culturing simpler and quicker – the process still takes days or even weeks, since typically, one or more days are required for the organism to grow in an initial enrichment broth, another day for a subsequent agar subculture, and at least another day to identify any colonies obtained by biochemical analysis.

In this communication, we sought to simplify the culture method, acknowledging the fact that culturing, though cumbersome, is indispensable for some diseases, and for others, it complements serology very well. Since it would be difficult to speed up the growth of an organism, we focused on the subsequent steps of identifying the organism after it is grown. As model disease, we chose enteric fever, a century-old disease that still poses a global health threat today. It is actually comprised of typhoid fever and the paratyphoid (type A, B or C) fevers. Typhoid fever, the most important, affects about 2 million people annually while paratyphoid A fever, which is clinically indistinguishable from typhoid, has recently emerged to be just as dangerous [5], [6]. These diseases are caused by different members of the *Salmonella* family. There are in fact over 2,000 members or serotypes of *Salmonella*, identified by the surface “O” and “H” antigens found in the lipopolysaccharide (LPS) and flagella of the organism, respectively [7]. Serotypes with a common immunodominant “O” antigen form a serogroup. Thus, O9 (and the structurally-juxtaposed O12 antigen) are found in serogroup D, which comprises *Salmonella* enterica serovar Typhi (*S*. Typhi), the cause of typhoid fever, and several other members that do not cause typhoid but instead, a self-limiting, gut-associated disease called gastroenteritis (commonly known as food poisoning). Of the serotypes that cause paratyphoid fever, O2 and O12 are found in *S*. Paratyphi A, O4 and O12 in *S*. Paratyphi B, while O6 and O7 are borne by *S*. Paratyphi C. Thus, O12 is common to *S.* Typhi, *S.* Paratyphi A and *S.* Paratyphi B. All other serotypes do not cause enteric fever but gastroenteritis rather. Recently, however, some of these serotypes, particularly *S*. Typhimurium (O4, O12) and *S*. Enteritidis (O9, O12), were found to invade the bloodstream of sub-Saharan African patients more frequently than *S*. Typhi or other types of bacteria, causing a new severe febrile illness called nontyphoidal salmonellosis (NTS) [8], [9].

**Figure 1:**
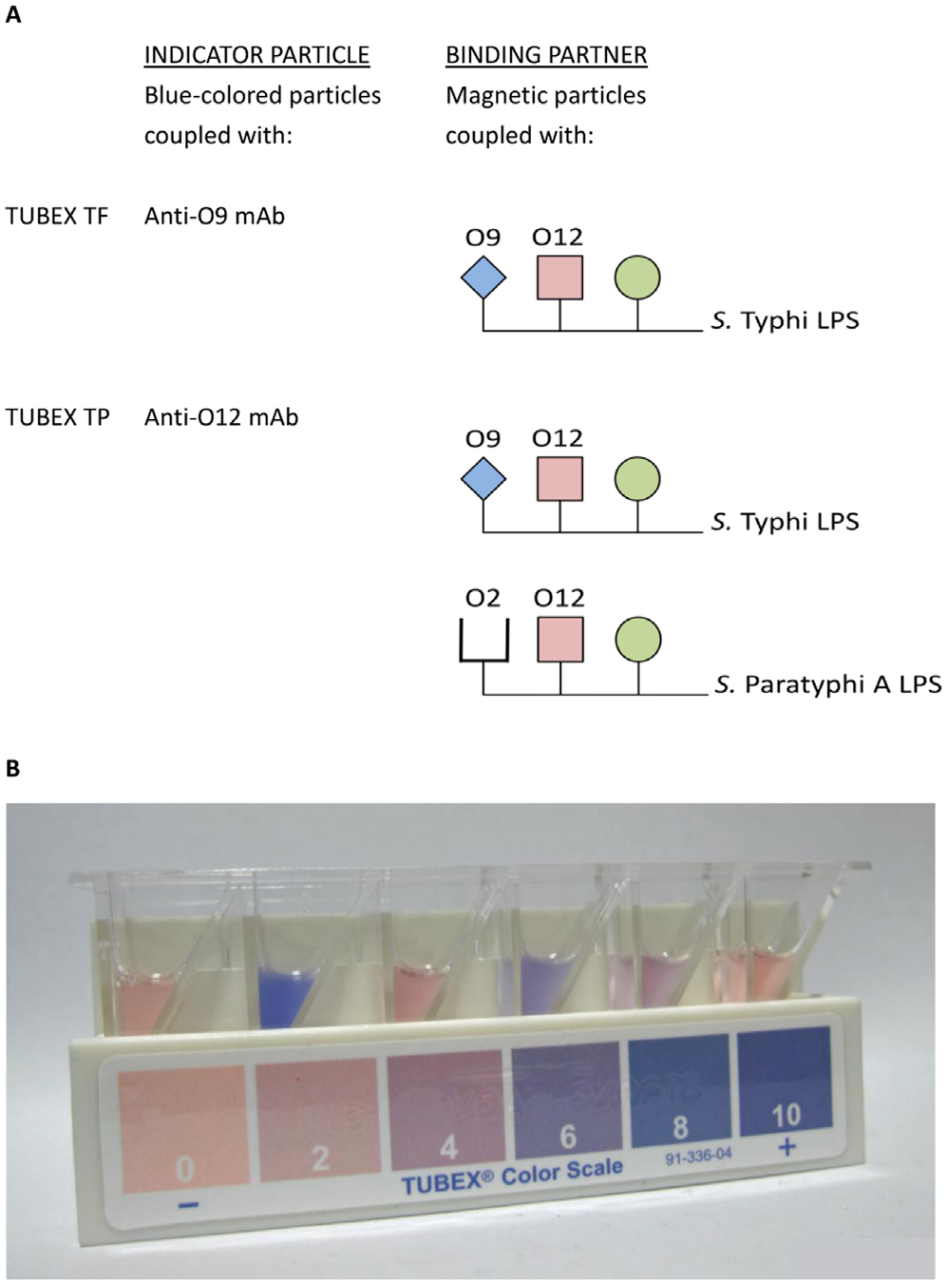
Principle and practice of TUBEX. Shown in (A) are the types of mAb and LPS utilized in TUBEX TP and TUBEX TF, and (B) pictorial representations of various TUBEX reactions performed in a set of reaction wells, placed on a magnet stand (Color Scale).

Both culture and serological methods are used to diagnose enteric fever, although culturing is not usually performed at small, peripheral laboratories in many countries. The organisms isolated by culture are characterized by phage-typing, antibiotic sensitivity testing and genome analysis, information that is extremely useful for epidemiology. Of the serological methods, the Widal test based on bacterial agglutination is the earliest, which is still widely used today. Many of the newer tests, which include TUBEX TF (IDL Biotech, Sweden) and lateral-flow tests [10], can be used at point-of-care settings and have fast turnaround times. TUBEX TF uniquely utilizes an inhibition assay format to detect anti-O9 antibodies from typhoid patients based on the ability of these antibodies to block the specific binding between a pair of microspheres. One of the particles (indicator) is blue-colored and coated with an O-9 specific monoclonal antibody (mAb), while the other is magnetic and coated with *S*. Typhi LPS. By virtue of the inhibition assay format, the test can also detect antigen (including whole bacteria) via blockade of the antibody-combining sites on the indicator particles. A red background color is added so that results are semi-quantitatively read after 5–10 min, based on the varying tones of blue and red in the supernatant: Most blue being most positive (see Fig. 1). Based on several studies, particularly ones conducted recently that had used ELISA as an objective benchmark, the TUBEX TF antibody-test was found in general to be both sensitive and specific for typhoid fever [11], [12].

We recently produced a prototypic TUBEX test of a different specificity using an anti-O12 mAb probe (Fig. 1). This was developed as a combined test to detect the common anti-O12 antibodies produced in both typhoid and paratyphoid A patients. In a recent evaluation, this test (TUBEX TP) proved, indeed, to be highly efficacious in detecting both diseases [4], but more interestingly, it detected typhoid patients better than TUBEX TF especially in terms of TUBEX scores. Herein, we applied TUBEX TP to the detection of whole organisms of *S.* Typhi and *S. *Paratyphi A grown as routine blood cultures (or as agar colonies) derived from enteric fever patients. We found excellent sensitivity and specificity with the test, attesting to the potential usefulness of immunological aids in simplifying microbiological procedures.

## Materials and Methods

### Study Cohort

Seventy-eight individuals (ages 3.5 to 18 yrs, median = 10 yrs) were recruited from “pediatric” outpatients (≤18 yrs) who visited the local primary healthcare clinics (*puskesmas*), the Ramelan Naval Hospital, and the Dr. Soetomo Hospital, all in Surabaya, Indonesia, between May 2010 and July 2011. For convenience, adult patients (≥19 yrs) were excluded from the study. The recruits were all residents in various suburbs of Surabaya, all had presented with high, persistent fever for at least the past 3 days (mean = 4.6 days) at the time of the visit, and none had sought prior medical attention. From each patient, blood was obtained by venipuncture and sent immediately to the Clinical Pathology Laboratory in Dr. Soetomo Hospital for microbiological investigation. (Written consent from patients was obtained, and the study protocol was approved by the local Ethics Committee in Surabaya, Indonesia [Komite Etik Penelitian Kesehatan Rsud Dr. Soetomo Surabaya].).

### Blood Culture

The routine blood culture method based on the protocol used by Clinical & Laboratory Standards Institute (CLSI), Pennsylvania, USA, and adopted by Dr. Soetomo Hospital, Surabaya, was followed. Three technical staff were involved. Briefly, blood (3.5 mL) obtained from each subject was inoculated into ox bile-containing culture broth (Oxoid, Basingstoke, Harts, UK; 35 mL). The broth was incubated aerobically at 37°C, and after 2 and 4 days (i.e. Day 3 and Day 5, respectively), the broth was sub-cultured (streaked) on *Salmonella-Shigella* (SS) agar plates (Pronadisa, Conda, Madrid, Spain). Colonies were identified using a set of conventional biochemical tests and also, in the case of *Salmonella* isolates, by slide agglutination using polyclonal antisera to *Salmonella* O-9, d-H, O-2 (PA) and O-4 (PB) (Biofarma, Bandung, Indonesia).

### Bacterial Detection from Blood Culture Broth

A small vol (1.0 ml) of the broth was dispensed into a microtube and centrifuged at 11,000 *g* for 5 mins in a micro-centrifuge (Microfuge 22R, Beckman Coulter, Brea, CA) (This clarification step removes the color of the broth.). The supernatant was removed by decanting, and any liquid remaining was removed using a piece of tissue paper (without disturbing the pellet). The pellet thus obtained was resuspended in 100 µl normal saline (0.9% NaCl). The suspension was transferred to a small glass tube and heated briefly (<2 mins) over a naked (kerosene) flame; heating was performed by quickly rotating the tube over the flame and, sometimes, withdrawing from it, to avoid over-heating or boiling. The treated suspension was then used in the TUBEX test, performed by a technical staff (FM) unconnected with culturing.

For cases that were TUBEX-positive, the unheated saline suspension was examined by slide agglutination using polyclonal antisera to *Salmonella* O-9, d-H, O-2 (PA) and O-4 (PB). For some cases (Patient nos. 52–78, Table S1), a glass slide smear was prepared from the unheated saline suspension and Gram-stained, and later viewed under high-power light microscopy.

### Bacterial Detection from SS Agar Colonies

Several colonies of the same morphological type were randomly selected from the SS agar plate and re-suspended in 100 µl normal saline in a small glass tube. Although suspect *Salmonella* colonies are non-lactose fermenting, for the purpose of specificity-testing in the study, lactose-fermenters were also examined. The suspension was heated briefly as described above, and similarly used in the TUBEX test. Slide agglutination was similarly used to identify *S*. Typhi, *S*. Paratyphi A and *S*. Paratyphi B organisms.

### TUBEX Tests

Two types of TUBEX tests were used: TUBEX TP, prepared as described previously [4], and TUBEX TF, obtained from IDL Biotech, Bromma, Sweden. Both tests were used in the same manner essentially as follows:

The Blue reagent (antigen-coated indicator particles) was dispensed in 50 µl vol to individual V-shaped chambers of a 6-chamber Reaction Well strip. The unknown sample (25 µl), comprising the broth- or colony-derived bacterial suspension, was then added to the Blue reagent and quickly mixed with it by pipetting the solution up and down several times. After 2 min, the Brown reagent (antigen-coated magnetic particles, 25 µl) was added to the mixture and the whole Reaction Well strip was sealed with adhesive tape, before being shaken rapidly for 2 mins in an automatic shaker (TUMIX, IDL Biotech). The set of reaction wells was then stood on the magnet stand provided, and after 2 mins, the results were read based on the resultant color of the supernatant. The results were read by eye and scored against the color chart provided: Score 0 (most red) – to – score 2 (faint red) were considered negative, and score 3 (faint blue) – to – score 10 (most blue) as increasingly positive.

## Results

We first investigated the capability of TUBEX TP to detect *S*. Typhi and *S*. Paratyphi A organisms in a preliminary study using bacterial strains isolated recently from our laboratory. The bacterial suspension obtained from organisms grown overnight in broth was examined in TUBEX TP. Of 2 *S*. Typhi and 8 *S*. Paratyphi A strains, all were found positive (TUBEX score 4–10), while a single strain of *E*. *coli* and *Pseudomonas aeruginosa* were both negative (TUBEX score 0) (data not shown). At the limit of detection (TUBEX score 4), the bacterial concentration was roughly 10^8^–10^9^ organisms/ml (equivalent to McFarland standard no. 3).

Next, we undertook a prospective study to determine the efficacy of TUBEX TP in the detection of *S*. Typhi and *S*. Paratyphi A organisms directly from routine blood cultures of 78 young outpatients suspected of having typhoid fever. The results are presented in Table S1 and summarized in Table 1. Thus, of the 78 cases, 18 (23.1%) grew *S*. Typhi, and 10 (12.8%) grew *S*. Paratyphi A. These results were determined by conventional culture and identification of the organisms grown from the blood broth after 2 days (Day 3) or 4 days (Day 5) of culture. (Earlier sampling times were not used.) Of the *S*. Typhi isolates, 83.3% (15/18) were obtained on Day 3, whereas in contrast, only 40.0% (4/10) of the *S*. Paratyphi A isolates were obtained at this early time-point.

**Table 1:** Summary of TUBEX TP performance in detecting *S*. Typhi and *S*. Paratyphi A organisms from routine blood culture broth.

		Day 3 broth+		Day 5 broth	
Organism isolated	Total no. cases	No. (%) found	TUBEX results*	No. (%) found	TUBEX results*
(a) *S*. Typhi	18	15 (83.3)	Positive (6–10)	3 (16.7)	Positive (8)
(b) *S*. Paratyphi A	10	4 (40.0)	Positive (4–10)	6 (60.0)	Positive (8)
(a)–(b)	28	19 (67.9)	Positive	9 (32.1)	Positive
(c) *E*. *coli*	12	8 (66.7)	Negative (0)	4 (33.3)	Negative (0)
(d) *Alcaligenes* spp.	1	1 (100)	Negative (0)		
(e) *Enterobacter* spp.	1	1 (100)	Negative (0)		
(c)–(e)	14	10 (71.4)	Negative	4 (28.6)	Negative
(f) None (no growth)	36			36 (100)	Negative (0)

+ After 2 days’ culture.

* Numericals indicate range of TUBEX scores.

TUBEX TP was performed on the Day 3 or Day 5 broth by a technical staff unconnected with microbiological culture, prior to the culture results being known. Bacterial saline suspension made from the broth was used; in some cases (Patient nos. 52–78, Table S1), a Gram-stained smear made of the suspension was examined microscopically, and in those broths that were later confirmed by SS agar culture to contain bacteria, large numbers of Gram-negative bacilli were revealed. As shown, the TUBEX TP results correlated 100% with the culture results. That is, (a) TUBEX TP was 100% sensitive in detecting *S*. Typhi and *S*. Paratyphi A organisms from blood culture, (b) 83.8% of *S*. Typhi organisms were detected after 2 days’ culture, the rest after 4 days, and (c) 40.0% of *S*. Paratyphi A were detected after 2 days, the rest after 4 days. TUBEX TP does not differentiate between *S*. Typhi and *S*. Paratyphi A. For such differentiation, after the TUBEX results were found positive, the bacterial saline suspension was examined by routine slide agglutination using antisera specific for *S*. Typhi (“O” and “H”), *S*. Paratyphi A (“PA”, O-specific) and *S*. Paratyphi B (“PB”, O-specific). In all cases, the identification of *S*. Typhi and *S*. Paratyphi A by slide agglutination agreed with the corresponding identification of the isolate by conventional culture (100% correlation). Of particular note is the absence of *S*. Paratyphi B organisms.

In 14 cases (14/78 or 17.9%), a non-*Salmonella* enteric bacillus was grown, namely, *E. coli* (12 cases), *Enterobacter* spp. (1) and *Alcaligenes* spp. (1). All these cases were also negative in TUBEX TP.

No organism grew on the SS agar after 6 days of broth culture for the rest of the cohort i.e. 36 cases (46.2%) were culture-negative. All these cases were also negative in TUBEX TP. Thus, altogether for the whole cohort, TUBEX TP was 100% specific (50/50).

In the same study involving the 78 patients, we also investigated whether TUBEX TP could directly identify colonies of *S*. Typhi and *S*. Paratyphi A grown on SS agar (subcultured from the blood culture broth). Saline suspension of the colonies was prepared and examined by TUBEX TP prior to the conventional biochemical identification of the colonies. The results displayed in Table S2 revealed that, indeed, TUBEX TP was 100% sensitive in detecting both *S*. Typhi (18 isolates) and *S*. Paratyphi A (10), and 100% specific with respect to the non-*Salmonella* isolates, namely, *E. coli* (12), *Alcaligenes* spp. (1) and *Enterobacter* spp. (1). Similar to broth culture, differentiation between *S*. Typhi and *S*. Paratyphi A was achieved by slide agglutination. As expected, 83.8% of *S*. Typhi isolates were identified by TUBEX TP on Day 4, the rest on Day 6, while 40.0% of *S*. Paratyphi A were identified on Day 4 and the rest on Day 6.

Finally, since TUBEX TP by itself cannot differentiate between *S*. Typhi and *S*. Paratyphi A organisms, we investigated whether TUBEX TF – which detects the O9 antigen but not O12– can be used as a complementary or differential test to single out the *S*. Typhi organisms. Thus, overnight broth cultures of S. Typhi (6 laboratory strains) and *S*. Paratyphi A (6 laboratory strains) were obtained and examined in both TUBEX TP and TUBEX TF. As shown in Table S3, whereas TUBEX TP detected both types of organisms in all cases (100% sensitive for both *S*. Typhi and *S*. Paratyphi A), TUBEX TF was positive only for *S*. Typhi (100% sensitive, 100% specific).

## Discussion

The present study demonstrates the feasibility of using immunological tools to simplify or shorten microbiological procedures – specifically, in this case, the use of antibodies to detect or identify *S*. Typhi and *S*. Paratyphi A organisms directly from broth or agar cultures. The reliability of this short-cut approach undoubtedly depends on the antibody probe used, as well as the method of application. Identification should in fact be regarded as presumptive unless the particular probe-method combination adopted has been proven through extensive evaluation to be truly specific for the organism in question. In a proven case, the risk of misidentification will be no greater than that associated with ordinary immunoassays that detect infections via the antibodies or soluble antigens found in the patient.

Two antibody probes highly specific for *Salmonella* were employed in our study; both are mAbs specific for unusual monosaccharides found in the LPS of the organism – the O9 and O12 antigens. If, for the sake of argument, polyclonal antibodies (antisera) are utilized instead of mAbs – as normally the case with slide agglutination tests (whether using soluble antibodies or antibodies bound on latex particles or *Staphylococcus* bacteria) – the test can theoretically become less reliable due to batch-batch variation of the antisera used, and less specific, because sub-specificities of anti-O12 antibodies (discovered recently [4]) could be present which recognize sub-epitopes that are less ‘private’ or restricted than others.

The method in which the antibody probe is used is important. Thus, whereas the TUBEX test can be used with confidence for the direct detection of *Salmonella* organisms from cultures, there is some reservation using the slide agglutination test in this regard (although, as discussed later, when used as a differential or secondary test, the demands are less stringent). A significant difference between slide agglutination (using soluble antibodies) and TUBEX is the indicator of reaction employed. In TUBEX, this is comprised of quality-controlled colored particles that are easy to visualize, whereas in slide agglutination, this is the dull-colored bacterial suspension prepared ad hoc from each selected culture. TUBEX results are thus more easily read and with less subjectivity. Another difference is: The inhibition assay format used in TUBEX avoids the potential non-specific reactivity due to environmental factors such as pH, or interfering substances, suffered by slide agglutination tests (regardless of type) [13]. In addition, the use of magnetic force to separate bound from unbound indicator particles in TUBEX ensures a quicker and cleaner read-out of the results compared to the slide tests.

Slide agglutination tests had in fact been used in the late 80 s to detect *Salmonella* and *Shigella* bacteria from stool enrichment broths, including one based on Vi detection [14], with mixed success [15]–[17] and little progress since then. A more successful application of these tests is the detection of other types of bacteria and their antigens from the cerebrospinal fluid of meningitis patients [18], [19]. Other immunological methods have also been experimented with in the past for whole bacteria or antigen detection, including immuno-diffusion, in which the organism is allowed to grow on antibody-embedded agarose [20], but most are cumbersome or time-consuming to perform.

In the present study, TUBEX TP was found to be 100% accurate in detecting *S*. Typhi and *S*. Paratyphi A organisms from routine blood culture broths and SS agar colonies. The TUBEX results agreed totally with those obtained by the traditional identification method. (Note, however, the clinical sensitivity of the culture method is a different matter – see later. Also, the TUBEX positive scores of 4– to –10 have no clinical significance other than merely denoting the bacterial concentration of the suspension made.) Although the study is small and has other limitations, including the notable absence of *Salmonella* serotypes other than *S*. Typhi and *S*. Paratyphi A, nonetheless, the findings confirm the potential of the immunodiagnostic approach that we first revealed in an experimental study [21]. In this study, TUBEX TF (O9-specific) was used to detect various laboratory strains of bacteria grown as broth cultures. As expected, the test was positive for 19 *Salmonella* serogroup D (O9^+^) strains, including 13 *S*. Typhi and 6 *S*. Enteritidis, and negative for 8 *Salmonella* isolates belonging to other (O9^−^) serotypes (7 *S*. Paratyphi A, 1 *S*. Typhimurium), as well as 2 *E. coli* strains. Interestingly, TUBEX TF was negative for an isolate previously identified by traditional methods to be *S*. Typhi; a re-examination using API 20E biochemical analysis and slide agglutination tests revealed the identity to be actually *S*. Typhimurium.

TUBEX TP casts a wider net in capturing *Salmonella* organisms than TUBEX TF, and hence, may be ideally suited for the screening of blood and stool cultures. A positive TUBEX TP suggests the presence of a *Salmonella* organism belonging to serogroup A, B or D. TUBEX TF can then be used as a rapid differential test to narrow this range since this test detects only serogroup D organisms. As demonstrated in the study, further differentiation can then be achieved quickly and simply by slide agglutination using appropriate ‘O’ and ‘H’ antisera. It is worth noting, however, that an isolate which is TUBEX TP-positive but TF-negative includes not only *S*. Paratyphi A, but also *S*. Paratyphi B, another paratyphoid organism that was not isolated in the present study but which can be found, albeit infrequently, in Surabaya (Nugraha J. et al, unpublished observations). Another candidate is *S*. Typhimurium, which has become increasingly important to public health as it accounted for 75% of the NTS bacteremia seen in febrile patients from Malawi, Africa [22]; in the same study, *S*. Enteritidis (both TUBEX TP- and TF-positive in theory) accounted for 21%.

Thus, TUBEX TP may serve as a useful adjunct to conventional culture since it can save at least 2 days the time needed to identify an isolate conventionally, including significant savings in costs and labor. A negative TUBEX test is just as useful as a positive test. Admittedly, even with the help of TUBEX TP, the whole process still took 2 days to detect (only) 80% of *S*. Typhi organisms in the present study. This, however, can theoretically be shortened to just a day for a first time-point in future, even though some sensitivity might be sacrificed. It is possible, too, that the culture-TUBEX approach be adopted by small, peripheral laboratories that are only minimally equipped – just to grow blood specimens in a clear broth medium (using a non-colored ox-gall substitute to obviate the need for centrifugation) in a 37°C water-bath or incubator. The culture broth can then be examined not by agar culture and biochemical identification, but periodically, with TUBEX TP by non-specialist personnel.

Several points of microbiological interest can be gleaned from the present study. First, the combined detection (isolation) of *S*. Typhi and *S*. Paratyphi A accounts for 35.9% (28/78) of the total blood cultures from patients suspected of having “typhoid” fever. However, if the isolates comprising of *E. coli*, *Alcaligenes* spp. and *Enterobacter* spp. are excluded, then the detection rate becomes 43.8% (28/64). This still includes the 36 cases considered as “no enteric bacilli found” which exhibited an absence of growth on SS agar; such cases, however, could be genuine typhoid but in whom few or no organisms prevailed in the circulation (e.g. in late-phase disease or due to prior antibiotic therapy), or these could be febrile infections due to *Staphylococcus* or certain viruses (but not dengue virus, since this was specifically excluded by routine serology), or parasites such as those causing malaria or typhus fever. Realistically, if we assume half of these “no growth” cases to be true typhoid, the success rate of microbiological culture for “typhoid” fever in the locality of our study (Surabaya, Indonesia), which is endemic for typhoid, becomes 60.9% (28/46). This is consistent with the generally poor isolation of *S*. Typhi organisms from blood by other investigators [23], [24]. However, in the above computation, *S*. Typhi is grouped together with *S*. Paratyphi A when the latter is actually responsible for quite a distinct disease. Leaving out *S*. Paratyphi A from the calculation, the actual detection of typhoid cases becomes a mere 39.1% (18/46). Clinically, thus, the diagnosis of “Typhoid” should be more appropriately termed “Enteric Fever” when referring to cases with high persistent fever and other symptoms characteristic of typhoid.

Second, the present findings document for the first time the unexpected high prevalence of paratyphoid A fever in Surabaya; very few cases were observed previously. The relative rate of isolation (roughly, 1∶2) of *S*. Paratyphi A vs *S*. Typhi is similar to those found previously in another part of Indonesia [25] and in Nepal [26], [27]. Paratyphoid A fever has indeed emerged recently as a global threat and is likely to have been mis- or under-diagnosed in many parts of the world still. The apparent low incidence in Surabaya seen previously may be due to an over-reliance on the Widal tests for diagnosis; indeed, we have hitherto seen very few PA-O positive cases compared to TO-positives (Nugraha J. et al, unpublished observations).

Third, from the majority (83.3%) of patients, *S*. Typhi was isolated early from the broth culture (after 2 days’ incubation), but interestingly in contrast, more (60%) *S*. Paratyphi A isolates were found later, after 4 days. An intriguing possibility is that *S*. Paratyphi A organisms normally circulate in low numbers in the patient which can thus explain, in part, the under-detection of these organisms. Some support for this is seen in the real-time PCR study of Nga et al [3], who found relatively lower copy numbers of target DNA sequence in the blood of patients for *S*. Paratyphi A compared to *S*. Typhi (39 vs 60 copies per ml), both being substantially lower than that found for *S*. Typhi in bone marrow (600 per ml).

The immunodiagnostic approach described herein can be used to detect viruses in culture or from body fluids, and is particularly useful in the food industry or public health. As an example, if enterotoxigenic *E. coli* is found to be the cause of an outbreak of food poisoning, and if bean sprouts are the prime suspect of the contamination, antibodies highly specific for the organism can then be used in a TUBEX test to quickly check the presence of the organism in enrichment broths of the sprouts obtained from different markets or gardens. By not having to grow the organism on agar (all the time), this can save invaluable time and effort in tracking down the contamination. Likewise, TUBEX TP may serve as a useful rapid test to screen dairy products for *Salmonella* organisms, although coverage will be improved if a pan-*Salmonella* (O) antibody is available as probe. Alternatively, antibodies directed against the flagella or fimbriae may serve the very purpose [28].

## Supporting Information

Table S1 **Rapid detection of** ***S*** **. Typhi and** ***S*** **. Paratyphoid A organisms from routine blood culture broth by TUBEX TP.**
(DOCX)

Table S2 **Rapid detection of** ***S*** **. Typhi and** ***S*** **. Paratyphoid A organisms from SS agar colonies by TUBEX TP.**
(DOCX)

Table S3 **Difference between TUBEX TP and TUBEX TF in the detection of** ***S*** **. Typhi and** ***S*** **. Paratyphi A organisms from known broth cultures.**
(DOCX)
