# Supplementary material for: Microbiological Culture Simplified Using Anti-O12 Monoclonal Antibody in TUBEX Test to Detect Salmonella Bacteria from Blood Culture Broths of Enteric Fever Patients
Source: PLoS One. 2012 Nov 16;7(11):e49586. doi: 10.1371/journal.pone.0049586 (PMC3500315; doi:10.1371/journal.pone.0049586)
Supplement: Table S1 — Rapid detection of S . Typhi and S . Paratyphoid A organisms from routine blood culture broth by TUBEX TP. (DOCX) [file pone.0049586.s001.docx]

Table S1. Rapid detection of *S*.Typhi and *S*.Paratyphi A organisms from routine blood culture broth by TUBEX TP.

| Patient No. | Age (years) | Sex | Duration of fever (days) | Day 3 broth | Day 3 broth:  Slide agglutination test | | | | Day 4 SS agar | Day 5 broth | Day 5 broth: Slide agglutination test | | | | Day 6 SS agar | Diagnosis by  traditional culture  (No. days) |
| --- | --- | --- | --- | --- | --- | --- | --- | --- | --- | --- | --- | --- | --- | --- | --- | --- |
|  |  |  |  | TUBEX score | O | H | PA | PB |  | TUBEX score | O | H | PA | PB |  |  |
| 1 | 15 | M | 5 | ND | ND | ND | ND | ND | NG | 0 | ND | ND | ND | ND | LF | *E*.coli (7) |
| 2 | 9 | M | 3 | ND | ND | ND | ND | ND | NG | 8 | - | - | + | - | NLF | *S*.Paratyphi A (7) |
| 3 | 16 | M | 3 | ND | ND | ND | ND | ND | NG | 8 | + | + | - | - | NLF | *S*.Typhi (7) |
| 4 | 14 | M | 4 | ND | ND | ND | ND | ND | NG | 8 | + | + | - | - | NLF | *S*.Typhi (7) |
| 5 | 17 | M | 3 | ND | ND | ND | ND | ND | NG | 8 | - | - | + | - | NLF | *S*.Paratyphi A (7) |
| 6 | 3.5 | M | 4 | 0 | ND | ND | ND | ND | NG | 0 | ND | ND | ND | ND | NG | Non-enteric |
| 7 | 6 | M | 7 | 0 | ND | ND | ND | ND | NG | 0 | ND | ND | ND | ND | NG | Non-enteric |
| 8 | 8 | F | 4 | 0 | ND | ND | ND | ND | NG | 0 | ND | ND | ND | ND | NG | Non-enteric |
| 9 | 18 | F | 7 | 0 | ND | ND | ND | ND | NG | 0 | ND | ND | ND | ND | NG | Non-enteric |
| 10 | 5 | M | 5 | 0 | ND | ND | ND | ND | NG | 0 | ND | ND | ND | ND | NG | Non-enteric |
| 11 | 4 | F | 4 | 0 | ND | ND | ND | ND | NG | 0 | ND | ND | ND | ND | NG | Non-enteric |
| 12 | 18 | M | 3 | 0 | ND | ND | ND | ND | NG | 0 | ND | ND | ND | ND | NG | Non-enteric |
| 13 | 4 | M | 3 | 0 | ND | ND | ND | ND | NG | 0 | ND | ND | ND | ND | NG | Non-enteric |
| 14 | 5 | F | 4 | 0 | ND | ND | ND | ND | NG | 0 | ND | ND | ND | ND | NG | Non-enteric |
| 15 | 7 | M | 5 | 0 | ND | ND | ND | ND | NG | 0 | ND | ND | ND | ND | NG | Non-enteric |
| 16 | 10 | M | 5 | 10 | - | - | + | - | NLF | ND | ND | ND | ND | ND | ND | *S*.Paratyphi A (5) |
| 17 | 10 | F | 3 | ND | ND | ND | ND | ND | NG | 8 | - | - | + | - | NLF | *S*.Paratyphi A (7) |
| 18 | 17 | M | 5 | 0 | ND | ND | ND | ND | NG | 8 | - | - | + | - | NLF | *S*.Paratyphi A (7) |
| 19 | 13 | M | 5 | ND | ND | ND | ND | ND | NG | 8 | + | + | - | - | NLF | *S*.Typhi (7) |
| 20 | 18 | M | 3 | ND | ND | ND | ND | ND | NG | 0 | ND | ND | ND | ND | NG | Non-enteric |
| 21 | 7 | F | 4 | ND | ND | ND | ND | ND | NG | 0 | ND | ND | ND | ND | NG | Non-enteric |
| 22 | 1 | M | 4 | ND | ND | ND | ND | ND | NG | 0 | ND | ND | ND | ND | NG | Non-enteric |
| 23 | 6 | M | 7 | ND | ND | ND | ND | ND | NG | 8 | - | - | + | - | NLF | *S*.Paratyphi A (7) |
| 24 | 11 | F | 5 | ND | ND | ND | ND | ND | NG | 0 | ND | ND | ND | ND | NG | Non-enteric |
| 25 | 7 | M | 5 | ND | ND | ND | ND | ND | NG | 8 | - | - | + | - | NLF | *S*.Paratyphi A (7) |
| 26 | 5 | M | 7 | 0 | ND | ND | ND | ND | NG | 0 | ND | ND | ND | ND | NG | Non-enteric |
| 27 | 12 | M | 5 | 0 | ND | ND | ND | ND | NG | 0 | ND | ND | ND | ND | NG | Non-enteric |
| 28 | 17 | M | 5 | 0 | ND | ND | ND | ND | NG | 0 | ND | ND | ND | ND | NG | Non-enteric |
| 29 | 12 | M | 5 | 0 | ND | ND | ND | ND | NG | 0 | ND | ND | ND | ND | LF | *E*.coli (7) |
| 30 | 18 | M | 5 | 0 | ND | ND | ND | ND | NG | 0 | ND | ND | ND | ND | NG | Non-enteric |
| 31 | 5 | F | 5 | 0 | ND | ND | ND | ND | NG | 0 | ND | ND | ND | ND | NG | Non-enteric |
| 32 | 7 | M | 6 | 0 | ND | ND | ND | ND | NG | 0 | ND | ND | ND | ND | LF | *E*.coli (7) |
| 33 | 6 | M | 5 | 0 | ND | ND | ND | ND | NG | 0 | ND | ND | ND | ND | NG | Non-enteric |
| 34 | 12 | F | 4 | 0 | ND | ND | ND | ND | NG | 0 | ND | ND | ND | ND | NG | Non-enteric |
| 35 | 14 | M | 3 | 0 | ND | ND | ND | ND | NG | 0 | ND | ND | ND | ND | NG | Non-enteric |
| 36 | 8 | F | 4 | 0 | ND | ND | ND | ND | NG | 0 | ND | ND | ND | ND | NG | Non-enteric |
| 37 | 14 | F | 5 | 0 | ND | ND | ND | ND | NG | 0 | ND | ND | ND | ND | NG | Non-enteric |
| 38 | 6 | M | 7 | 0 | ND | ND | ND | ND | NG | 0 | ND | ND | ND | ND | NG | Non-enteric |
| 39 | 9 | M | 4 | 0 | ND | ND | ND | ND | NG | 0 | ND | ND | ND | ND | NG | Non-enteric |
| 40 | 12 | M | 4 | 0 | ND | ND | ND | ND | NG | 0 | ND | ND | ND | ND | NG | Non-enteric |
| 41 | 10 | M | 5 | 0 | ND | ND | ND | ND | NG | 0 | ND | ND | ND | ND | NG | Non-enteric |
| 42 | 17 | M | 4 | 0 | ND | ND | ND | ND | NG | 0 | ND | ND | ND | ND | NG | Non-enteric |
| 43 | 13 | M | 5 | 0 | ND | ND | ND | ND | NG | 0 | ND | ND | ND | ND | NG | Non-enteric |
| 44 | 11 | F | 7 | 0 | ND | ND | ND | ND | NG | 0 | ND | ND | ND | ND | NG | Non-enteric |
| 45 | 9 | M | 5 | 0 | ND | ND | ND | ND | NG | 0 | ND | ND | ND | ND | NG | Non-enteric |
| 46 | 8 | M | 6 | 0 | ND | ND | ND | ND | NG | 0 | ND | ND | ND | ND | NG | Non-enteric |
| 47 | 8 | M | 5 | 10 | + | + | - | - | NLF | ND | ND | ND | ND | ND | ND | *S*.Typhi (5) |
| 48 | 4 | F | 5 | ND | ND | ND | ND | ND | NG | 0 | ND | ND | ND | ND | NG | Non-enteric |
| 49 | 5 | F | 4 | ND | ND | ND | ND | ND | NG | 0 | ND | ND | ND | ND | NG | Non-enteric |
| 50 | 3.5 | M | 6 | 0 | ND | ND | ND | ND | NG | 0 | ND | ND | ND | ND | NG | Non-enteric |
| 51 | 15 | M | 5 | 0 | ND | ND | ND | ND | LF | 0 | ND | ND | ND | ND | ND | *E*.coli (5) |
| 52 | 12 | F | 4 | 0 | ND | ND | ND | ND | LF | 0 | ND | ND | ND | ND | ND | *E*.coli (5) |
| 53 | 16 | M | 3 | 0 | ND | ND | ND | ND | LF | 0 | ND | ND | ND | ND | ND | *E*.coli (5) |
| 54 | 5 | M | 3 | 0 | ND | ND | ND | ND | LF | 0 | ND | ND | ND | ND | ND | *E*.coli (5) |
| 55 | 10 | F | 5 | 0 | ND | ND | ND | ND | LF | 0 | ND | ND | ND | ND | ND | *Enterobacter* spp. (5) |
| 56 | 12 | F | 4 | 0 | ND | ND | ND | ND | LF | 0 | ND | ND | ND | ND | ND | *E*.coli (5) |
| 57 | 11 | F | 4 | 0 | ND | ND | ND | ND | LF | 0 | ND | ND | ND | ND | ND | *E*.coli (5) |
| 58 | 8 | M | 3 | 0 | ND | ND | ND | ND | NG | 0 | ND | ND | ND | ND | NLF | *Alkaligenes* spp. (5) |
| 59 | 10 | F | 4 | 0 | ND | ND | ND | ND | NG | 0 | ND | ND | ND | ND | LF | *E*.coli (5) |
| 60 | 18 | F | 3 | ND | ND | ND | ND | ND | NG | 0 | ND | ND | ND | ND | LF | *E*.coli (7) |
| 61 | 17 | M | 7 | 4 | - | - | + | - | NLF | ND | ND | ND | ND | ND | ND | *S*.Paratyphi A (5) |
| 62 | 15 | M | 6 | 10 | + | + | - | - | NLF | ND | ND | ND | ND | ND | ND | *S*.Typhi (5) |
| 63 | 7 | M | 6 | 10 | + | + | - | - | NLF | ND | ND | ND | ND | ND | ND | *S*.Typhi (5) |
| 64 | 17 | M | 5 | 10 | + | + | - | - | NLF | ND | ND | ND | ND | ND | ND | *S*.Typhi (5) |
| 65 | 4.5 | F | 6 | 8 | + | + | - | - | NLF | ND | ND | ND | ND | ND | ND | *S*.Typhi (5) |
| 66 | 5 | F | 7 | 8 | + | + | - | - | NLF | ND | ND | ND | ND | ND | ND | *S*.Typhi (5) |
| 67 | 16 | M | 5 | 8 | + | + | - | - | NLF | ND | ND | ND | ND | ND | ND | *S*.Typhi (5) |
| 68 | 6 | M | 5 | 8 | + | + | - | - | NLF | ND | ND | ND | ND | ND | ND | *S*.Typhi (5) |
| 69 | 12 | F | 6 | 8 | + | + | - | - | NLF | ND | ND | ND | ND | ND | ND | *S*.Typhi (5) |
| 70 | 15 | F | 5 | 8 | + | + | - | - | NLF | ND | ND | ND | ND | ND | ND | *S*.Typhi (5) |
| 71 | 14 | F | 4 | 0 | ND | ND | ND | ND | LF | 0 | ND | ND | ND | ND | ND | *E*.coli (5) |
| 72 | 13 | M | 5 | 6 | - | - | + | - | NLF | ND | ND | ND | ND | ND | ND | *S*.Paratyphi A (5) |
| 73 | 9 | M | 7 | 8 | + | + | - | - | NLF | ND | ND | ND | ND | ND | ND | *S*.Typhi (5) |
| 74 | 16 | M | 5 | 6 | + | + | - | - | NLF | ND | ND | ND | ND | ND | ND | *S*.Typhi (5) |
| 75 | 13 | F | 5 | 8 | - | - | + | - | NLF | ND | ND | ND | ND | ND | ND | *S*.Paratyphi A (5) |
| 76 | 7 | F | 5 | 8 | + | + | - | - | NLF | ND | ND | ND | ND | ND | ND | *S*.Typhi (5) |
| 77 | 10 | M | 6 | 8 | + | + | - | - | NLF | ND | ND | ND | ND | ND | ND | *S*.Typhi (5) |
| 78 | 5.5 | F | 4 | 8 | + | + | - | - | NLF | ND | ND | ND | ND | ND | ND | *S*.Typhi (5) |

F,female; M, male; NG, no growth; LF, lactose-fermenter; NLF, non-lactose fermenter; Non-enteric, no enteric bacilli isolated; Slide agglutination test performed on un-heated pellet suspension; ND, not done

Nb.

1. Pellet suspension from Day 3 or Day 5 broth in 27 patients (Patient # 52 to Patient # 78) was also Gram-stained and examined by high-power microscopy, which revealed large numbers of Gram-negative bacilli in all cases.
2. Pellet suspension from Day 3 or Day 5 broth in 18 patients not included in above list (7 female, 11 male; age 0.5 -17 yr, median 7.33 yr; fever duration 3-6 days, median 4.33 days) was similarly Gram-stained and examined by high-power microscopy, but no organism was seen and TUBEX thus not performed.
