# Supplementary material for: Microbiological Culture Simplified Using Anti-O12 Monoclonal Antibody in TUBEX Test to Detect Salmonella Bacteria from Blood Culture Broths of Enteric Fever Patients
Source: PLoS One. 2012 Nov 16;7(11):e49586. doi: 10.1371/journal.pone.0049586 (PMC3500315; doi:10.1371/journal.pone.0049586)
Supplement: Table S2 — Rapid detection of S . Typhi and S . Paratyphoid A organisms from SS agar colonies by TUBEX TP. (DOCX) [file pone.0049586.s002.docx]

Table S2. Rapid detection of *S*.Typhi and *S*.Paratyphi A organisms from SS agar colonies by TUBEX TP.

| Patient No. | Growth  SS agar  (Day 4) | Growth SS agar  (Day 6) | TUBEX score (Day 4 or 6) | Biochemical tests (KIA ) | | | | Other biochemical tests | | | | Slide agglutination tests | | | | Diagnosis by traditional culture  (No. days) |
| --- | --- | --- | --- | --- | --- | --- | --- | --- | --- | --- | --- | --- | --- | --- | --- | --- |
|  |  |  |  | Slant | Butt | H_2_S | Gas | Cit | Mot | Ind | Urea | O | H | PA | PB |  |
| 1 | NG | LF | 0 | acid | acid | - | + | - | + | + | - | ND | ND | ND | ND | *E*.coli (7) |
| 2 | NG | NLF | 10 | alkaline | acid | - | + | - | + | - | - | - | - | + | - | *S*.Paratyphi A (7) |
| 3 | NG | NLF | 10 | alkaline | acid | +weak | - | - | + | - | - | + | + | - | - | *S*.Typhi (7) |
| 4 | NG | NLF | 10 | alkaline | acid | + | - | - | + | - | - | + | + | - | - | *S*.Typhi (7) |
| 5 | NG | NLF | 10 | alkaline | acid | - | + | - | + | - | - | - | - | + | - | *S*.Paratyphi A (7) |
| 16 | NLF | ND | 10 | alkaline | acid | - | + | - | + | - | - | - | - | + | - | *S*.Paratyphi A (5) |
| 17 | NG | NLF | 10 | alkaline | acid | - | + | - | + | - | - | - | - | + | - | *S*.Paratyphi A (7) |
| 18 | NG | NLF | 10 | alkaline | acid | - | + | - | + | - | - | - | - | + | - | *S*.Paratyphi A (7) |
| 19 | NG | NLF | 10 | alkaline | acid | +weak | - | - | + | - | - | + | + | - | - | *S*.Typhi (7) |
| 23 | NG | NLF | 10 | alkaline | acid | - | + | - | + | - | - | - | - | + | - | *S*.Paratyphi A (7) |
| 25 | NG | NLF | 10 | alkaline | acid | - | + | - | + | - | - | - | - | + | - | *S*.Paratyphi A (7) |
| 29 | NG | LF | 0 | acid | acid | - | + | - | + | + | - | ND | ND | ND | ND | *E*.coli (7) |
| 32 | NG | LF | 0 | acid | acid | - | + | - | + | + | - | ND | ND | ND | ND | *E*.coli (7) |
| 47 | NLF | NG | 10 | alkaline | acid | +weak | - | - | + | - | - | + | + | - | - | *S*.Typhi (5) |
| 51 | LF | ND | 0 | acid | acid | - | + | - | + | + | - | ND | ND | ND | ND | *E*.coli (5) |
| 52 | LF | ND | 0 | acid | acid | - | + | - | + | + | - | ND | ND | ND | ND | *E*.coli (5) |
| 53 | LF | ND | 0 | acid | acid | - | + | - | + | + | - | ND | ND | ND | ND | *E*.coli (5) |
| 54 | LF | ND | 0 | acid | acid | - | + | - | + | + | - | ND | ND | ND | ND | *E*.coli (5) |
| 55 | LF (cream-  colored) | ND | 0 | acid | acid | - | + | - | + | + | - | ND | ND | ND | ND | *Enterobacter* spp. (5) |
| 56 | LF | ND | 0 | acid | acid | - | + | - | + | + | - | ND | ND | ND | ND | *E*.coli (5) |
| 57 | LF | ND | 0 | acid | acid | - | + | + | + | - | + | ND | ND | ND | ND | *E*.coli (5) |
| 58 | LF  (opaque, adherent) | ND | 0 | alkaline | alkaline | - | + | - | + | + | - | ND | ND | ND | ND | *Alkaligenes* spp.  (5) |
| 59 | LF | ND | 0 | acid | acid | - | + | - | + | + | - | ND | ND | ND | ND | *E*.coli (5) |
| 60 | NLF | ND | 0 | acid | acid | - | + | - | + | + | - | ND | ND | ND | ND | *E*.coli (7) |
| 61 | NLF | ND | 4 | alkaline | acid | - | + | - | + | - | - | + | - | + | - | *S*.Paratyphi A (5) |
| 62 | NLF | ND | 10 | alkaline | acid | +weak | - | - | + | - | - | + | + | - | - | *S*.Typhi (5) |
| 63 | NLF | ND | 10 | alkaline | acid | +weak | - | - | + | - | - | + | + | - | - | *S*.Typhi (5) |
| 64 | NLF | ND | 10 | alkaline | acid | +weak | - | - | + | - | - | + | + | - | - | *S*.Typhi (5) |
| 65 | NLF | ND | 8 | alkaline | acid | +weak | - | - | + | - | - | + | + | - | - | *S*.Typhi (5) |
| 66 | NLF | ND | 8 | alkaline | acid | +weak | - | - | + | - | - | + | + | - | - | *S*.Typhi (5) |
| 67 | NLF | ND | 6 | alkaline | acid | +weak | - | - | + | - | - | + | + | - | - | *S*.Typhi (5) |
| 68 | NLF | ND | 8 | alkaline | acid | +weak | - | - | + | - | - | + | + | - | - | *S*.Typhi (5) |
| 69 | NLF | ND | 8 | alkaline | acid | +weak | - | - | + | - | - | + | + | - | - | *S*.Typhi (5) |
| 70 | NLF | ND | 6 | alkaline | acid | +weak | - | - | + | - | - | + | + | - | - | *S*.Typhi (5) |
| 71 | LF | ND | 0 | acid | acid | - | + | - | + | + | - | ND | ND | ND | ND | *E*.coli (5) |
| 72 | NLF | ND | 8 | alkaline | acid | - | + | - | + | - | - | + | - | + | - | *S*.Paratyphi A (5) |
| 73 | NLF | ND | 6 | alkaline | acid | +weak | - | - | + | - | - | + | + | - | - | *S*.Typhi (5) |
| 74 | NLF | ND | 8 | alkaline | acid | +weak | - | - | + | - | - | + | + | - | - | *S*.Typhi (5) |
| 75 | NLF | ND | 8 | alkaline | acid | - | + | - | + | - | - | + | - | + | - | *S*.Paratyphi A (5) |
| 76 | NG | NLF | 8 | alkaline | acid | + | - | - | + | - | - | + | + | - | - | *S*.Typhi (5) |
| 77 | NLF | ND | 8 | alkaline | acid | +weak | - | - | + | - | - | + | + | - | - | *S*.Typhi (5) |
| 78 | NLF | ND | 8 | alkaline | acid | +weak | - | - | + | - | - | + | + | - | - | *S*.Typhi (5) |

Patient no. refers to same individual as in Table S1; Cit, citrate; Mot, motility; Ind, indol; NG, no growth; LF, lactose-fermenter; NLF, non-lactose fermenter; +,positive reaction; -, negative reaction; Slide agglutination test performed on KIA slant culture; ND, not done
