# Supplementary material for: Microbiological Culture Simplified Using Anti-O12 Monoclonal Antibody in TUBEX Test to Detect Salmonella Bacteria from Blood Culture Broths of Enteric Fever Patients
Source: PLoS One. 2012 Nov 16;7(11):e49586. doi: 10.1371/journal.pone.0049586 (PMC3500315; doi:10.1371/journal.pone.0049586)
Supplement: Table S3 — Difference between TUBEX TP and TUBEX TF in the detection of S . Typhi and S . Paratyphi A organisms from known broth cultures. (DOCX) [file pone.0049586.s003.docx]

Table S3. Difference between TUBEX TP and TUBEX TF in the detection of *S*. Typhi and *S*. Paratyphi A organisms from known broth cultures.

| *S*. Typhi isolate | TUBEX TP | TUBEX TF |
| --- | --- | --- |
| ST1 | 8 | 8 |
| ST2 | 6 | 8 |
| ST3 | 6 | 8 |
| ST4 | 6 | 8 |
| ST5 | 8 | 8 |
| ST6 | 8 | 8 |
| *S*. Paratyphi A isolate |  |  |
| SPA1 | 6 | 0 |
| SPA2 | 8 | 0 |
| SPA3 | 10 | 0 |
| SPA4 | 8 | 0 |
| SPA5 | 8 | 0 |
| SPA6 | 10 | 0 |

Results expressed as TUBEX scores
